# Gradient Microstructures and Mechanical Properties of Ti-6Al-4V/Zn Composite Prepared by Friction Stir Processing

**DOI:** 10.3390/ma12172795

**Published:** 2019-08-30

**Authors:** Yuting Lv, Zihao Ding, Xueyan Sun, Lei Li, Gang Sha, Rui Liu, Liqiang Wang

**Affiliations:** 1 College of Mechanical and Electronic Engineering, Shandong University of Science and Technology, Qingdao 266590, Shandong, China; 2 State Key Laboratory of Metal Matrix Composites, School of Materials Science and Engineering, Shanghai Jiao Tong University, 800 Dongchuan Road, Shanghai 200240, China; 3 Key Laboratory of Unconventional Oil & Gas Development, China University of Petroleum (East China), Qingdao 266590, Shandong, China; 4 Herbert Gleiter Institute of Nanoscience, Nanjing University of Science and Technology, Nanjing 210094, China; 5 National Engineering Research Center for Nanotechnology (NERCN), 28 East JiangChuan Road, Shanghai 200241, China

**Keywords:** friction stir processing, TC4/Zn, microstructures, composite

## Abstract

In this work, a biomedical Ti-6Al-4V (TC4)/Zn composite with gradient microstructures was successfully prepared by friction stir processing (FSP). The microstructures and mechanical properties of the composite were systematically studied using scanning electron microscope (SEM), X-ray diffractometer (XRD), transmission electron microscope (TEM), atom probe tomography (APT), and microhardness test. The results show that TC4/Zn composite can be successfully prepared, and gradient microstructures varying from coarse grain to nanocrystalline is formed from the bottom to the upper surface. During FSP, adding Zn can accelerate the growth of β phase region, and the grain size significantly increases with the increasing rotation rate. The grain combination is the main mechanism for grain growth of β phase region. The deformation mechanisms gradually change from dislocation accumulations and rearrangement to dynamic recrystallization from the bottom to the upper surface (1.5 mm–150 μm from the upper surface). The composite exhibits slightly higher microhardness compared with the matrix. This paper provides a new method to obtain a TC4/Zn composite with gradient surface microstructures for potential applications in the biomedical field.

## 1. Introduction

Due to the high strength to weight ratio, high corrosion resistance, and biocompatibility, Ti-6Al-4V (TC4) alloys have attracted extensive attention in the field of biomedical implant materials [1,2]. For bone implants, the service life of the orthopedic and dental implants is significantly affected by the interaction between the implant material and the tissue [3,4,5]. On one hand, wear debris caused by friction between the implant and tissue may lead to side effects for the organism, such as cytotoxicity, inflammation, anaphylaxis, and so on [6,7]. On the other hand, TC4 alloy has a higher Young’ s modulus than that of bone, which can lead to “stress shielding” [8,9]. In addition, surface biological compatibility is also important for bone implants, which directly affect early cell adhesion, while cellular adhesion is the primary stage of the interaction of tissue and implants. Thus, it is highly desirable to increase the surface mechanical properties and simultaneously improve biological compatibility of TC4 alloy [10,11]. 

In the past few years, extensive efforts have been made to improve the surface mechanical properties of Ti alloys [12,13]. It is well acknowledged that severe plastic deformation (SPD), such as friction stir processing (FSP), accumulative roll-bonding (ARB) [14], and equal-channel angular pressing (ECAP) [15,16], is a feasible method to obtain a Ti alloy with a high hardness and a low elastic modulus [17,18]. Li et al. [2] attributed the added hardness of FSPed TC4 alloy to the refined β-regions with ultra-fine grained α phase. Wang et al. [19] reported that the grain size of Ti-35Nb-2Ta-3Zr alloy can be reduced to nanoscale, which significantly enhances its surface hardness. Among all the SPD methods, FSP is a relatively new surface modification technique, which has received increasing attention due to its energy efficiency, environment friendliness, and versatility [17]. The grain of metal or alloy can be significantly refined during FSP due to severe plastic deformation and simultaneously elevating deformation temperature [20,21]. 

Zn is an important element in the human body, due to its role in the synthesis of a variety of enzymes, improving bone growth, and maintaining cell physiological function [22,23]. However, there are significant differences between the melting point of Ti (1660 °C) and the boiling point of Zn (907 °C), and, thus, it is difficult to add Zn into a Ti alloy via conventional metallurgical processing [24,25]. Brice et al. [24] successfully prepared Ti–Zn compound via a self-designed reaction chamber, and found that α-Ti has a much larger solubility than in the equilibrium phase diagram. Vassilev et al. [26] studied the reaction kinetics in the Ti–Zn system via the heat diffusion method. Yang et al. [27] investigated the Zn-rich corner of the Zn-Al-Ti system using a diffusion couple approach. However, it is difficult to simultaneously obtain better biocompatibility and mechanical properties via the aforementioned methods. While FSP is a semi-solid processing method, which has lower peak temperature. FSP can be used to add Zn into a TC4 alloy to obtain a surface TC4/Zn composite. Its feasibility lies in two aspects. First, there is a relatively lower peak temperature during FSP compared with conventional metallurgical methods. Second, the circulating flow of the material around FSP tool can realize homogeneous distribution of Zn element. 

In this paper, a novel TC4/Zn composite with gradient microstructures was prepared via adding Zn into a TC4 alloy by FSP. The microstructures of the composite including the effect of the added Zn on grain growth mechanisms of β phase region and spheroidization behavior of α lamellae in coarse grain were systematically studied using scanning electron microscope (SEM), transmission electron microscope (TEM), and atom probe tomography (APT), etc. 

## 2. Experimental

### 2.1. Fabrication of TC4/Zn Composite

**Figure 1:**
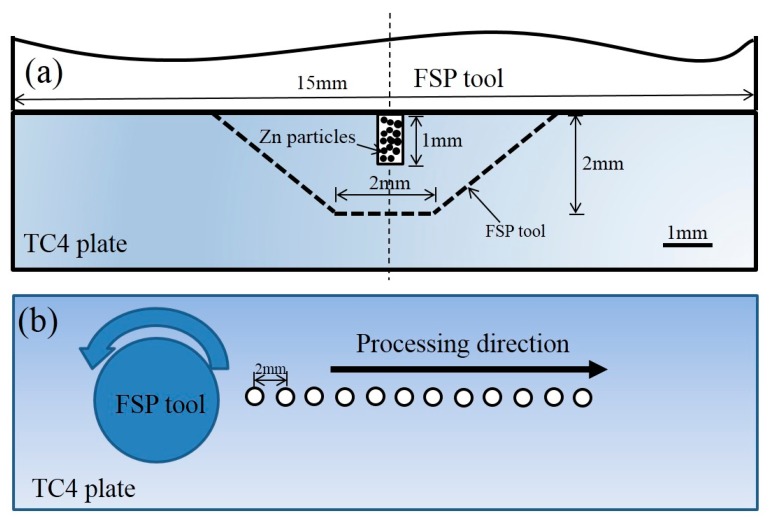
(**a**) Schematic diagrams showing the dimensions of the FSP tool and (**b**) the hole distribution in the TC4 plate.

The matrix used in this work is a commercial TC4 alloy plate with a thickness of 5 mm. The dimension of the FSP tool and hole distribution in the TC4 plate are shown in Figure 1. Commercial nano Zn powder with a particle size of ~50 nm was used to add into the TC4 alloy. The material of the FSP tool used in this work was a tungsten carbide (WC)-based alloy, and detailed tool size was reported in our previous work [1]. After the powder was loaded into holes, FSP was performed on the TC4 plate at the same traverse speed (υ) of 100 mm/min and at several rotation rates of 225 rpm, 300 rpm, and 375 rpm respectively. Accordingly, the composite prepared at different rotation rates were named as FSP225, FSP300, and FSP375 respectively.

### 2.2. Microstructural Observations

The microstructures in the stir zone of the composite with various processing parameters (~0.5 mm from the upper surface) were observed using a JEOL 7600-F field emission gun scanning electron microscope (SEM, JEM-2100F, Tokyo, Japan). For SEM sample preparation, a longitudinal section of the FSP stir zone was first cut from the FSPed TC4 plates using wire electrical discharge machining, and then ground, polished, and etched in a solution composed of 2 vol% HF, 6 vol% HNO_3_, and 92 vol% water for 5 s. Phase constituents were identified for the nanocomposite plate with a distance of 0.5 mm from the upper surface using a D8 ADVANCE X-ray diffractometer (XRD, D/max-III A, Tokyo, Japan) with Cu Kα radiation at room temperature. A JEM-200 EX transmission electron microscope (TEM, JTM-2100F, Tokyo, Japan) was used to observe microstructures in the whole FSP zone range from the upper surface to the bottom (0~1.5 mm). The atom probe tomography (APT, Beijing, China) was performed at Nanjing University of Science and Technology (Nanjing, China) and relative processing parameters were reported in previous investigation [28].

## 3. Results

### 3.1. Effects of Added Zn on Microstructures of TC4/Zn Composites

**Figure 2:**
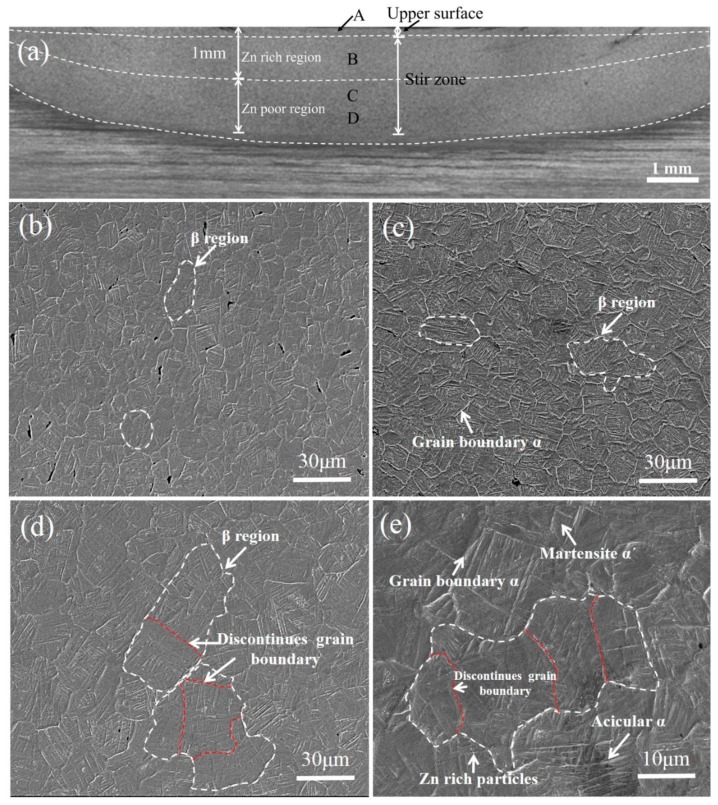
Optical microscopy (OM) and SEM micrographs showing the microstructures of TC4/Zn composites: (**a**) overview of different regions at low magnification (OM image). (**b**) FSP225; (**c**) FSP300; (**d**) FSP375 and (**e**) is a magnified version of Figure 2d.

**Figure 3:**
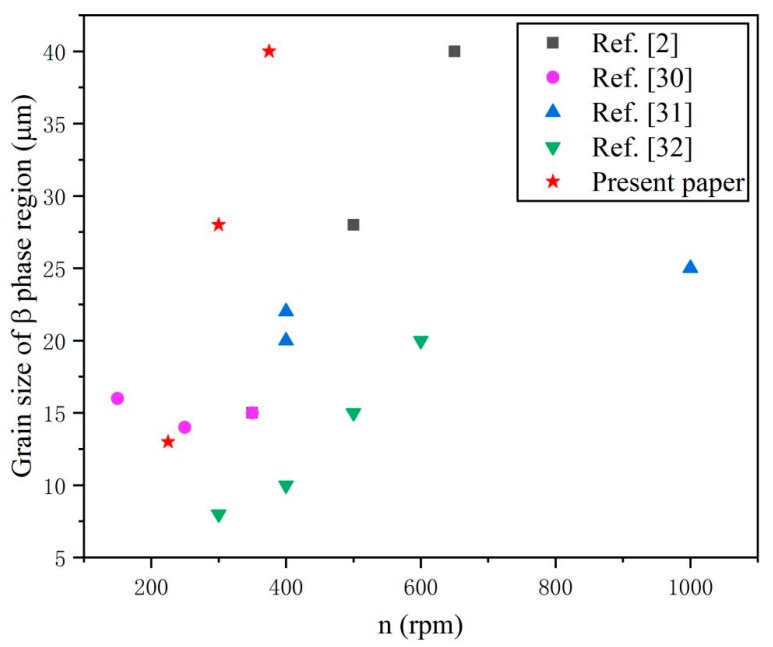
Grain size variation of β phase region in the FSPed TC4 alloy reported in previous investigations [2,30,31,32].

Figure 2a shows an overview of different regions at low magnification. In order to study the effect of added Zn on microstructures of TC4/Zn composites, the microstructures of the Zn-rich region (i.e., region B in Figure 2a) were observed. Figure 2b–e shows the SEM micrographs of TC4/Zn composites obtained at various processing parameters. As seen in Figure 2b–d, the microstructures of samples mainly contain β phase regions (indicated by white dashed line). Magnified images (not shown) clearly show that the composite displays similar microstructures to the composite prepared at a traverse speed of 50 mm/min and a rotation rate of 300 rpm, which has been reported in our previous investigation [29]. However, it should be noted that the grain size of β region in the TC4/Zn composite increases with the increasing rotation rate, which are 10~15, 25~30, and 35~45 μm for the FSP225, FSP300, and FSP375 samples, respectively (Figure 2b–d). In order to understand the effect of Zn on the grain growth of the composite, we compared the grain size of the composite in present work with many previous investigations [2,30,31,32], in which TC4 was processed by FSP without any additions. The summarized data are displayed in Figure 3. As can be seen from Figure 3, the composite obtained in the present work has a much bigger β phase region compared with the FSPed TC4 alloy under similar processing parameters, such as a higher rotation rate (400~1000 rpm). In Figure 3, it is also concluded that the grain size of the β phase region increases with an increasing rotation rate. Therefore, it is quite clear that adding Zn into a TC4 alloy using FSP can accelerate the growth of the β phase region. As seen in Figure 2e, discontinues grain boundaries are found in the FSP375 sample (shown by red dashed line). In many previous investigations [2,30,31,32], some discontinuous grain boundaries were observed in the FSPed TC4 alloy, indicating that FSP can also form discontinuous grain boundaries in a TC4 alloy without any additions. After adding Zn into the TC4 alloy, under similar processing condition, much more discontinuous grain boundaries were observed in the TC4/Zn composite. Thus, we believe that β grain combination is the main grain growth mechanism in the composite. When the TC4/Zn composite is subjected to severe plastic deformation, small grains gather together. The dislocation cells in some neighboring grains with small differences in the orientation angle transfer to other grain boundaries via dissociation or a disassembly effect, which results in the absence of some grain boundaries.

### 3.2. TEM Observations of Microstructural Variation with Decreasing Distance from the Upper Surface (150 μm–1500 μm)

**Figure 4:**
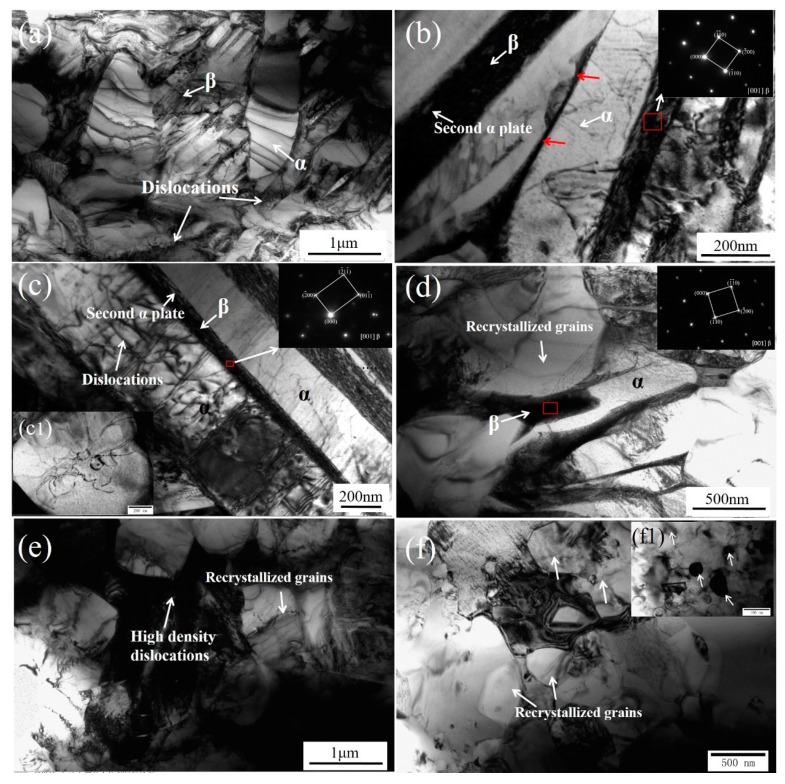
TEM images of the stir zone for FSP225 sample (about 0.5 mm~1.2 mm from the upper surface): The distances of (**a**), (**b**), (**c**), (**d**), (**e**) and (**f**) from upper surface gradually decrease. The Figure c1 shows the dislocation division in α grain, and the Figure f1 shows the Zn-rich particles in matrix.

Figure 4 shows TEM images of TC4/Zn composite prepared at the rotation rate of 225 rpm. Figure 4a is obtained at a distance of about 1.5 mm from the upper surface (i.e., region D in Figure 2a). It is clearly shown that the microstructure mainly consists of coarser α lamellae together with β phase laths, which is typical in α+β titanium alloy. Shear deformation in a thick α lamella was observed, and did not transmit across the adjacent β phase. Our previous investigations reported that there is a temperature gradient in the stir zone during FSP, and the temperature gradually decreases from upper surface to bottom [33]. The area is located in the bottom of the stir zone, thus there is relatively lower temperature. There is also a higher α phase content. In this case, the α phase bears most of the stress, and, thus, plastic deformation firstly occurs in α phase. With the decrease in distance from the upper surface, the content of the coarser α phase decreases and that of the β phase increases. As seen in Figure 4b, the shear deformation caused by stress considerably decreases. The dislocation density in α phase increases considerably. Some offsets in the interphase boundaries (see red arrow in Figure 4b) are clearly observed, indicating that a dislocation slip across the α/β interface occurs [34]. In region C of stir zone (Figure 2a), high-density dislocations and some localized dislocation bands are observed in α lamellae. These dislocations may accumulate and rearrange to split the coarser α phase. The illustration in Figure 4c,c1 also clearly shows that the α grain is divided by dislocations to form smaller grains. Thus, we believed that dislocation accumulations and rearrangement is the main mechanism for the fragmentation of α lamellae in this region. As the plastic deformation increases, dislocation tangles occur and dislocation walls are formed, leading to the fragmentation of α lamellae (Figure 4c). With a further increase of the deformation degree (i.e., region B in Figure 2a), α/β lamellae features gradually disappear and some α/β grain boundaries cannot be discerned easily (Figure 4d). Thereafter, more high-density dislocations areas are formed, in which α grains nucleate and dynamic recrystallization occurs (see Figure 4e,f). In addition, the distribution of Zn-rich particles can represent Zn distribution. The detailed TEM observation results show that there is a heterogeneous distribution from the upper surface to the bottom. The content of the Zn-rich particles gradually decreases from the upper surface to the bottom (See Figure 4f1). This is attributed to the large temperature gradient and strain difference from the upper surface to the bottom of stir zone.

### 3.3. X-ray Diffraction Pattern Analysis

**Figure 5:**
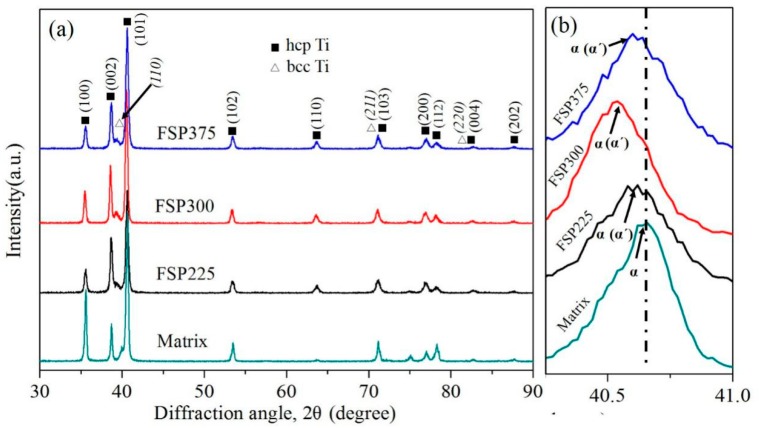
(**a**) XRD patterns for the specimens obtained under different rotation rates, and (**b**) Magnified image in (**a**) between 40° and 41°.

Figure 5a shows the XRD patterns of the specimens obtained under different rotation rates. As seen from Figure 5a, compared with the matrix TC4 alloy, there are no significant differences for the TC4/Zn composite. Figure 5b is the magnified XRD pattern of the composite prepared under different processing parameters. Compared with matrix alloy, the diffraction peak of α phase for the composite significantly shifted to a smaller 2θ angle. Malinov et al. [35] ascribed the widening or shift of the XRD pattern of the TC4 alloy in this location to the formation of martensite α’ phase. They considered the diffusion-less transformations of β→α’ in the TC4 alloy during rapid cooling results in microscopic volume expansion, which may influence the lattice parameters [2,35]. It should be noted that the composite obtained at the rotation rate of 300 rpm has a larger shift than those at 225 rpm and 375 rpm. In other words, more martensite α’ is formed in the FSP300 sample. TEM observations indicate that FSP225 contains more Zn-rich particles than FSP300 and FSP375 samples and the concentration of Zn-rich particles decreases with an increasing rotation rate. This is because increasing the rotation rate during FSP can result in a higher peak temperature of the composite, thereby incorporating more Zn into the α-Ti crystal structure to form a solid solution. On the other hand, Zn is a β-Ti eutectoid stabilizer with a relatively high eutectoid transformation temperature of ~620 °C, which may impede the formation of martensite α’ [29,36,37].

### 3.4. Microstructures in Upper Surface of TC4/Zn Composite (0–150 μm)

**Figure 6:**
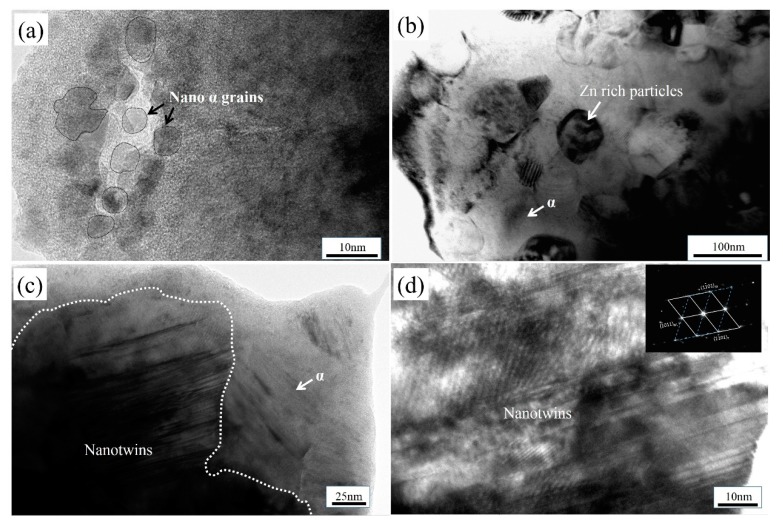
TEM images of TC4/Zn composite in the upper surface: (**a**) Nano α grains, (**b**) Zn rich particles, (**c**) Nano α twins, and (**d**) highly magnified image of nanotwins in Figure 6c and the inset showing the SAED pattern of the nanotwins.

Figure 6 shows the microstructure images of TC4/Zn composite (0–150 μm) formed in the upper surface. In Figure 6a, selected area electron diffraction (not shown) of the area shows that only α phase exists in this region. It is indicated that nano α grains with grain size of below 10 nm are formed in upper surface. The grain boundaries are marked by white dashed lines. It can be seen that the nanograins have relatively clear grain boundaries. Our previous investigation indicated that grain boundary sliding was a major deformation mechanism in nanograins with grain size of <10 nm [33]. In Figure 6b, Zn-rich particles with a grain size below 100 nm are also found. It was reported that α-Ti had a slightly larger solubility of the Zn element at Ti-rich side of the Ti–Zn phase diagram [24]. Therefore, it can be speculated that the added Zn can react with Ti to form Zn-rich particles or dissolve into the α matrix to form a solid solution during FSP. Nanotwins are also found in the upper surface of the composite, as indicated by the dashed lines in Figure 6c. The highly magnified image of nanotwins in Figure 6c shows clearly that the grains have twin morphologies and the inset confirms that the twins are the {$10\bar{1}1$}<$10\bar{1}\bar{2}$> twinning system, and only the twinning system is found in the TC4/Zn composite. The nanotwin system is formed via the dynamic overlapping of two partial dislocations [33].

### 3.5. APT Characterization

**Figure 7:**
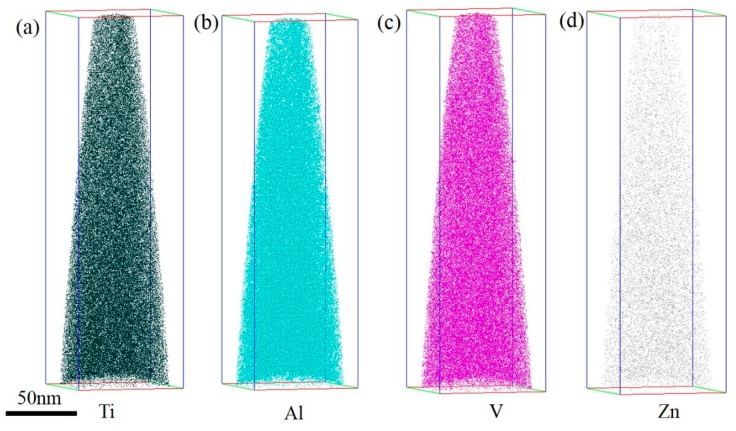
3DAP atoms mapping results of TC4/Zn composite: (**a**) Ti, (**b**) Al, (**c**) V and (**d**) Zn.

APT was used to analyze chemical compositions and their distributions in the upper surface of the TC4/Zn composite. The 3DAP atoms mapping is shown in Figure 7. The uniformly distributed Zn element was found in the composite matrix, although Zn has a lower solid solution in the Ti alloy. Zn-rich particles were also observed in the surface region (Figure 6b). Therefore, it is convincing that part of the added Zn reacts with Ti to form Zn-rich particles, and the other part dissolves into the α matrix to form a solid solution.

### 3.6. Microhardness

**Figure 8:**
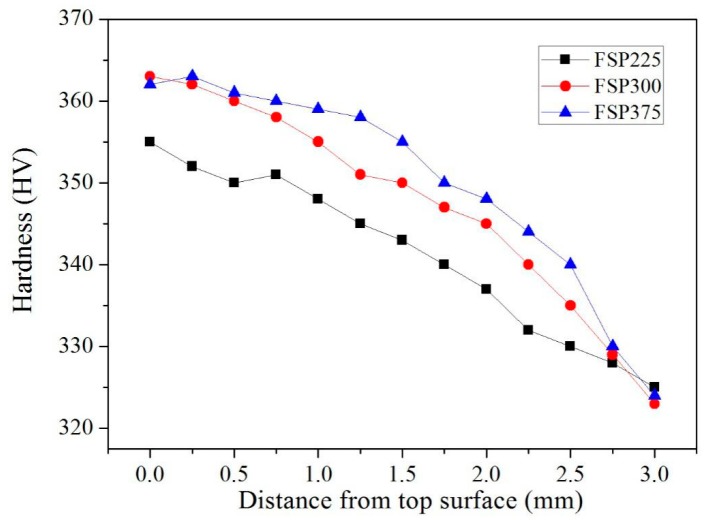
Microhardness distribution profile obtained from the top surface to bottom of TC4/Zn composite.

Figure 8 shows the microhardness of the TC4/Zn composite obtained from the top surface to bottom. Compared with the matrix, the TC4/Zn composite has a higher microhardness, and the microhardness increases with an increasing rotation rate. This is because during FSP the composite experiences severe plastic deformation, and the grains are significantly refined. The peak temperature also exceeds α/β phase transition temperature, and a fine flaky or acicular α phase can be formed following air cooling. These grain and phase boundaries can block dislocation motion, increasing the microhardness of the composite. Increasing the rotation rate can increase the strain of the composite in the stir zone, and, thus, the microhardness of the composite increases with an increasing rotation rate. In addition, part of the added Zn element was dissolved into the Ti matrix to form a solid solution. The others react with Ti to form Zn-rich particles. Thus, added Zn can also increase the microhardness of the composite via solid solution strengthening and grain refinement. It should be noted that the grain size of the β phase region increases with an increase of rotation rate due to the existence of liquid Zn. The β phase has a body-centered cubic structure, the microhardness of which is lower than that the α phase with a close-packed hexagonal structure. These have an adverse effect on the microhardness of the composite. That may be the reason why the microhardness has no significant increase with an increase in the rotation rate.

## 4. Discussion

### 4.1. Effect of Added Zn on β Grain Growth Mechanism During FSP

Microstructure observation results indicate that the grain size of the β phase region significantly increases with an increasing rotation rate (i.e., the plastic deformation) after adding Zn into the TC4 alloy. This is contrary to well-established knowledge in plastic deformation processing, which asserts that the grain size of alloys decreases with increasing plastic deformation [16,38,39]. As reported in many investigations, the grain growth behavior of titanium alloys mainly involves grain boundary migration controlled by atom diffusion [40,41]. In the present work, although FSP can form some discontinuous grain boundaries because of severe plastic deformation, more discontinuous grain boundaries are found in the FSP375 sample (Figure 2d,e). It is indicated that the grain combination is one of the main mechanisms for grain growth of the β phase region. As known to all, grain growth for an alloy depends on the driving force of the interface energy difference before and after deformation. During FSP, grain boundary sliding occurs due to the existence of liquid state Zn. There is a relatively higher interface energy in this case. In order to decrease the interface energy and make grain boundary sliding easier, some neighboring grains with small orientation angle differences transfer to other grain boundaries via a dissociation or disassembly effect, which can result in the disappearance of grain boundaries. In addition, TEM observations indicate that the FSP225 sample has more Zn-rich particles than the FSP300 and FSP375 samples and the content of Zn-rich particles decreases with the increase in rotation rate. Zn-rich particles with a grain size of a few tens of nanometers may inhibit the grain growth of the β phase via the Zener pinning effect. An analogous phenomenon has also been found in nickel aluminum bronze, in which a second phase with a grain size of tens of nanometers can inhibit grain boundary mobility by Zener pinning [42]. While an increasing rotation rate significantly decreases the amount of Zn-rich particles, the Zener pinning effect caused by these particles also obviously decreases. That may be another reason why there is such a big difference in the grain size between FSP225 and FSP375 samples (Figure 2b,d).

### 4.2. Spheroidization Behavior of α Lamellae Microstructure in Stir Zone

During FSP, the stir zone in the TC4/Zn composite is subjected to severe plastic deformation. In region D of stir zone (See Figure 4a), due to a lower peak temperature and smaller plastic deformation, the microstructures of the composite mainly consist of more numerous and coarser α lamellae, leading to the occurrence of plastic deformation firstly in the α grain. The plastic deformation and peak temperature simultaneously increase the closer they are to region C, which results in the formation of more β phases. It is apparent that the dislocation bands do not transmit across the α/β interface, which can lead to the stress concentration in α/β phase boundaries. A high-density dislocation wall and bands are also observed in the α lamellae. The illustration in Figure 4c clearly shows that the α grain is divided by dislocations to form smaller grains. This indicates that dislocations accumulate and rearrange in the α phase during FSP, and the mechanism plays an important role in the spheroidization of the α lamellae in this region. This is because due to lower peak temperature in this region, the mechanical effect may contribute more to the spheroidization of the α lamellae. As the local stress increases, dislocations accumulate in the α lamellae, and then recovery can occur by dislocation cross-slip, resulting in the disappearance of opposite sign dislocations on intersecting slip planes [43,44,45,46,47,48].

In region B (See Figure 2a), compared with region C, the plastic deformation and peak temperature simultaneously increase. The peak temperature in the stir zone can be very high because of the severe friction between the alloy and FSP tool, which can be higher than 1000 °C according to previous investigations [2,30,49]. Figure 4e,f clearly shows the dynamic recrystallization behavior of the α grain. Thus, in the region, the lamellar structure of the TC4 alloy is broken up via dynamic recrystallization, leading to the formation of equiaxed microstructures of α+β phase (Figure 4f). As plastic deformation increases, the recrystallized grains grow and, simultaneously, α/β boundaries rotate towards one another, leading to the formation of globularization microstructures [48]. Therefore, we believe that the deformation mechanisms change from dislocation accumulation and rearrangement in the lower area (region C and D in Figure 2a), to dynamic recrystallization in the higher area (region B in Figure 2a). Zherebtsov et al. [47] reported that continuous dynamic recrystallization of β layer occurs first, and then the lamellae are fragmented by the grooving/boundary splitting mechanism. For the α layer, the globularization mechanism is mainly associated with boundary splitting at higher temperatures (800 °C). The contribution of boundary splitting significantly decreases at relatively lower temperatures (600 °C) due to the greater thickness of the α lamellae.

## 5. Conclusions

In this study, a TC4/Zn composite with gradient surface microstructures was successfully fabricated by friction stir processing (FSP). Microstructures and mechanical properties of the composite were systematically studied by SEM, TEM, and APT. The following conclusions could be drawn:

1. Zn element can be added into a TC4 alloy via FSP. Part of the added Zn reacts with Ti to form Zn-rich particles, and the other part dissolves into the α matrix to form a solid solution. Gradient surface microstructures are also formed, which vary from coarse grain scale in the stir zone to nanoscale in the upper surface.

2. During FSP, added Zn can increase the grain size of the β phase region in the composite due to its lower melting point. The grain size of the β phase region also significantly increases with an increasing rotation rate. The grain combination is the main mechanism for the growth of β phase region.

3. In the stir zone of the composite (150 μm–1500 μm), the spheroidization mechanism of the α lamellae changes gradually from dynamic recrystallization of the α grain to a dislocation accumulation and rearranging mechanism, as the distance from the upper surface increases, accompanied by a decrease in deformation degree and peak temperature. Compared with matrix, the TC4/Zn composite has slightly higher microhardness.
